# Methylmalonic acidemia triggers lysosomal-autophagy dysfunctions

**DOI:** 10.1186/s13578-024-01245-1

**Published:** 2024-05-17

**Authors:** Michele Costanzo, Armando Cevenini, Laxmikanth Kollipara, Marianna Caterino, Sabrina Bianco, Francesca Pirozzi, Gianluca Scerra, Massimo D’Agostino, Luigi Michele Pavone, Albert Sickmann, Margherita Ruoppolo

**Affiliations:** 1 Department of Molecular Medicine and Medical Biotechnology, University of Naples Federico II, Via Pansini 5, Naples, 80131 Italy; 2 CEINGE–Biotecnologie Avanzate Franco Salvatore, Naples, Italy; 3 Leibniz-Institut für Analytische Wissenschaften – ISAS – e.V, Dortmund, Germany; 4 Department of Chemistry, College of Physical Sciences, University of Aberdeen, Aberdeen, Scotland, United Kingdom; 5 Medizinische Fakultät, Medizinische Proteom-Center (MPC), Ruhr-Universität Bochum, Bochum, Germany

**Keywords:** Methylmalonic acidemia, Lysosomes, Autophagy, Metabolic disease, Multi-omics, Multi-proteomics, MMA therapy

## Abstract

**Background:**

Methylmalonic acidemia (MMA) is a rare inborn error of propionate metabolism caused by deficiency of the mitochondrial methylmalonyl-CoA mutase (MUT) enzyme. As matter of fact, MMA patients manifest impairment of the primary metabolic network with profound damages that involve several cell components, many of which have not been discovered yet. We employed cellular models and patients-derived fibroblasts to refine and uncover new pathologic mechanisms connected with MUT deficiency through the combination of multi-proteomics and bioinformatics approaches.

**Results:**

Our data show that MUT deficiency is connected with profound proteome dysregulations, revealing molecular actors involved in lysosome and autophagy functioning. To elucidate the effects of defective MUT on lysosomal and autophagy regulation, we analyzed the morphology and functionality of MMA-lysosomes that showed deep alterations, thus corroborating omics data. Lysosomes of MMA cells present as enlarged vacuoles with low degradative capabilities. Notwithstanding, treatment with an anti-propionigenic drug is capable of totally rescuing lysosomal morphology and functional activity in MUT-deficient cells. These results indicate a strict connection between MUT deficiency and lysosomal-autophagy dysfunction, providing promising therapeutic perspectives for MMA.

**Conclusions:**

Defective homeostatic mechanisms in the regulation of autophagy and lysosome functions have been demonstrated in MUT-deficient cells. Our data prove that MMA triggers such dysfunctions impacting on autophagosome-lysosome fusion and lysosomal activity.

**Supplementary Information:**

The online version contains supplementary material available at 10.1186/s13578-024-01245-1.

## Introduction

Organic acidemias represent a wide and heterogeneous group of rare inherited metabolic disorders that affect amino acid and protein metabolism, with subsequent accumulation of organic acids that reach toxic concentrations in biofluids [1–3]. The most common group is represented by methylmalonic acidemias, with an estimated incidence of around 1:100,000 newborns in Europe, North America, Asia-Pacific, and the Middle East and North Africa regions [4].

In particular, the isolated methylmalonic acidemia (MMA; OMIM #251000) is due to deficiency of the methylmalonyl-CoA mutase (MUT) enzyme which, in association with its cofactor 5’-deoxyadenosylcobalamin [5], is involved in the catabolism of odd-chain fatty acids, cholesterol, valine, methionine, isoleucine and threonine. Impaired activity/expression of MUT enzyme due to mutations in the *MMUT* gene fails to isomerize methylmalonyl-CoA into succinyl-CoA in the mitochondrial matrix, with subsequent disruption of propionyl-CoA metabolism and mitochondrial damage. As a consequence of the loss of MUT enzymatic activity, methylmalonyl-CoA and its related acid (methylmalonic acid) accumulate, triggering structural and functional defects in diverse metabolic pathways and in mitochondrial energy network, which in turn lead to gross organ dysfunctions mainly impacting the brain, liver and kidney functions [6–8]. In addition, MMA is associated with secondary perturbations of propionyl-CoA metabolism that involve increased levels of propionylcarnitine, propionylglycine, 2-methylcitric acid, and propionic acid [9]. Acylcarnitines and organic acids are easily detectable and quantifiable in liquid biopsies (e.g. dried blood spots) by metabolomics approaches [10, 11]. Using liquid chromatography-tandem mass spectrometry (LC-MS/MS), these markers are dosed to perform diagnosis for MMA in newborns [12]. Hence, the pathogenesis of MMA relies on the accumulation of toxic metabolites and on secondary unbalances in energy production and mitochondrial defects generated by the inhibition of oxidative phosphorylation, intensification of oxidative stress and lowered antioxidant capacity [13].

Over the past years, the understanding of phenotype-genotype correlations in MMA has improved, and different circulating markers have been proposed to assess disease burden, monitor disease progression and therapeutic response [7], as patients who respond to administration of vitamin B12 have a more favorable prognosis. In fact, MMA patients are classified into two distinct clinical phenotypes depending on the complete or partial loss of MUT apoenzyme as mut0 or mut–, with only the latter having a partial response to vitamin B12. Patients who are non-responsive to vitamin B12 treatment (mut0) show more severe pathological complications that include metabolic acidosis, hyperammonemia, hyperglycinemia, ketosis and ketonuria, developmental delay, as well as liver dysfunction and kidney disease [8, 14]. These patients are often subjected to a liver or combined liver-kidney transplant, whereas managing their metabolic conditions with dietary treatment (restriction of propionigenic amino acids and carnitine supplementation) fails [15]. Nevertheless, renal or liver disease can continue progressing also post-transplantation [16, 17].

MMA is then a complex metabolic disorder, and the dysfunctions responsible for the systemic injury in MMA remain without mechanistic validation, although widely described. Our study expands the current understanding of MMA by integrating the results obtained from the most advanced state-of-the-art proteome and bioinformatic analyses with biochemical and imaging experiments. Our research highlights lysosomal and autophagic dysfunctions as new features of MMA, proposing to investigate and target lysosomal alterations to get mechanistic insights into the pathogenesis of this rare metabolic condition.

## Results

### Proteomic signature of the HEK 293 MUT-cell models

**Fig. 1:**
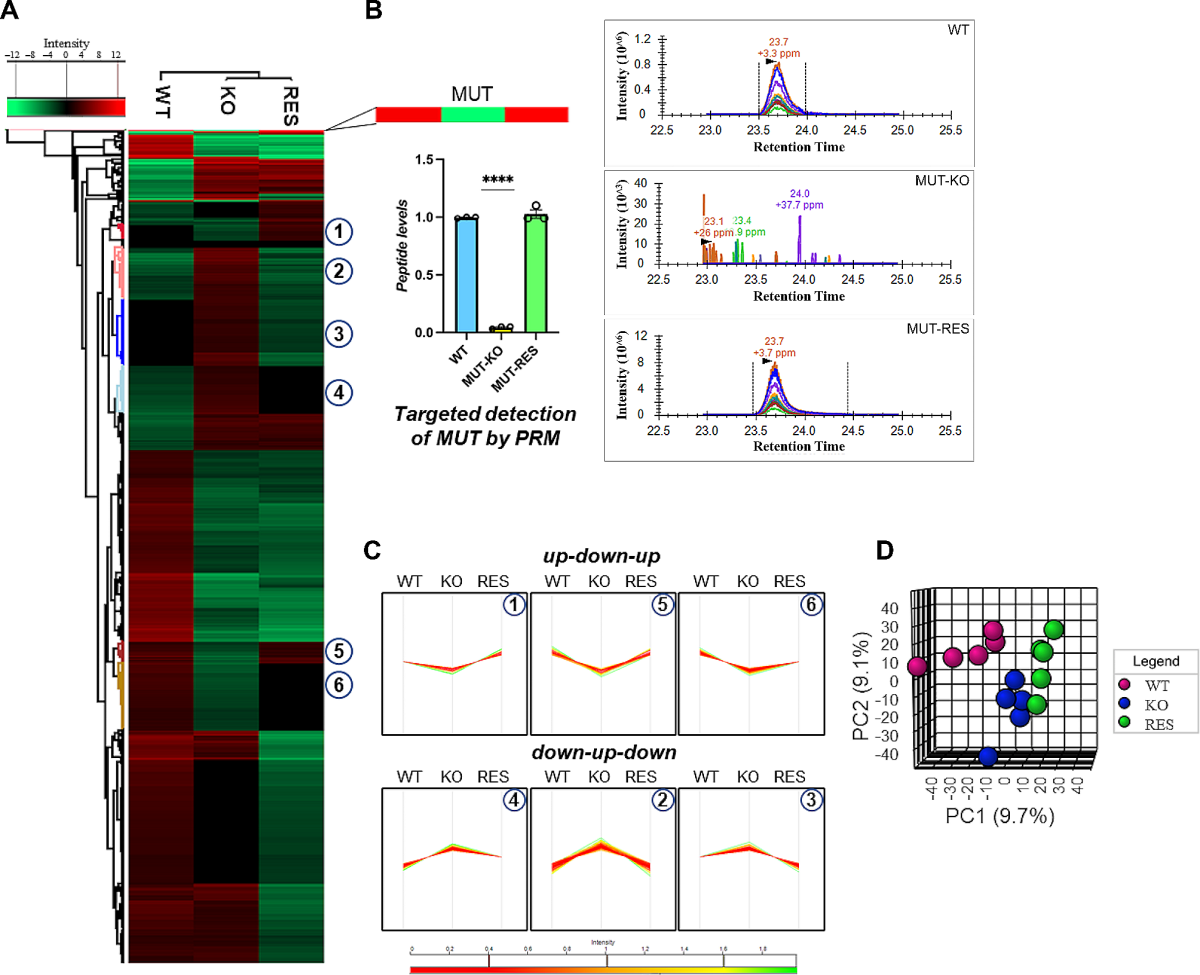
Analysis of the DIA-MS proteomic dataset of WT, MUT-KO, and MUT-RES cells. **(A)** Heatmap visualization of the proteins quantified in the three cell conditions, with detail of the abundance of MUT protein. **(B)** Representative chromatograms and bar plot of MUT peptides quantification revealed by targeted PRM analysis. Three experimental replicates were performed for statistical analysis, *p* < 0.0001. **(C)** Protein clusters selected from the heatmap and signed with circled numbers showing specific trends of abundance defined as up-down-up and down-up-down. **(D)** PCA analysis of the three experimental proteome datasets

In our previous work, we described the generation of a MMA HEK 293cellular model by CRISPR/Cas9-mediated knock-out of the *MMUT* gene (MUT-KO) [18]. Then, we rescued MUT expression with a vector encoding for a FLAG-*MUT* sequence (MUT-RES cells), to be used as specific control in addition to the wild-type (WT) cells. In the current work, the proteomes of HEK 293 WT, MUT-KO, and MUT-RES cell lines were analyzed by untargeted data-independent acquisition (DIA) proteomics, globally resulting in 4725 proteins correctly quantified with a minimum of one unique peptide in more than the 50% of the LC-MS/MS runs (*n* = 15) (Fig. 1A). Notably, MUT protein was the most down-regulated protein in the MUT-KO dataset compared to WT and MUT-RES samples, as expected. As additional validation, MUT peptides were targeted by PRM, confirming the absence of MUT-related ions in MUT-KO cells (Fig. 1B). The statistical comparison of the three cell conditions permitted to extract six clusters of proteins (Fig. 1A, C) in which the trend of abundance was decreased in MUT-KO and concomitantly increased in WT and MUT-RES cells (up-down-up, clusters 1, 5, 6), and vice versa (down-up-down, clusters 4, 2, 3). The PCA showed the statistical separation of the analyzed groups (Fig. 1D).

**Fig. 2:**
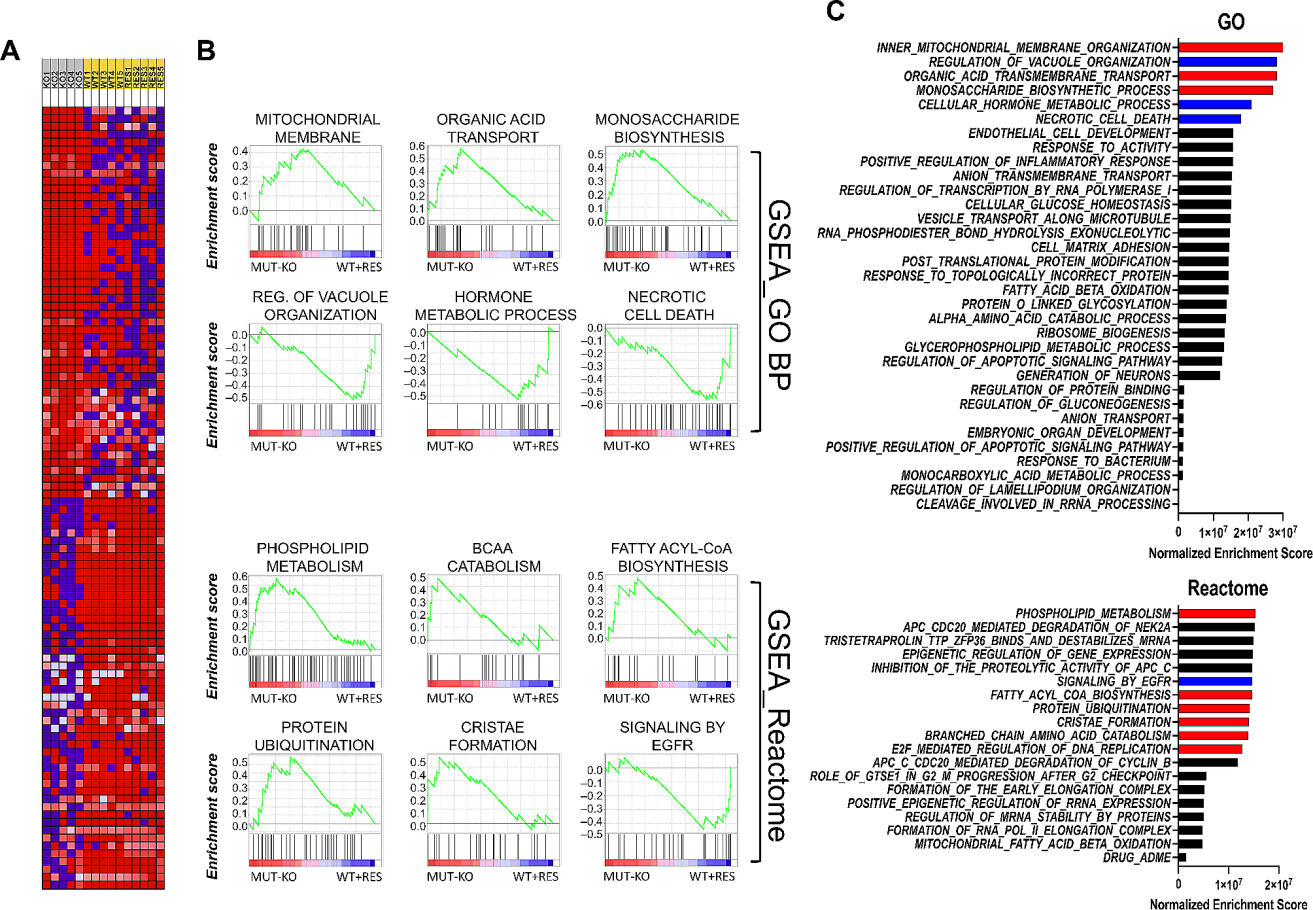
Gene Set Enrichment Analysis (GSEA) of the whole MUT-KO proteome. **(A)** Heatmap of the top-100 features showing correlation between the ranked genes and the phenotypes (MUT-KO vs. WT + RES). Expression values are represented by range of colors from red to blue corresponding to high and low abundances, respectively. **(B)** GSEA analysis enriched significant GO BP and Reactome terms. The densest hits (highest normalized enrichment score) correlating with the MUT-KO were selected and plotted from **(C)** the total list of retrieved terms. Red and blue bars represent positively and negatively correlated terms, respectively. Black bars represent terms that may be significant but redundant or irrelevant

To pinpoint quantitative alterations of the MUT-KO proteome, Gene Set Enrichment analysis (GSEA) of the whole proteome dataset was performed comparing the MUT-KO against the WT plus MUT-RES control conditions (WT + RES). Using the Gene Ontology Biological Process (GO BP) and Reactome gene sets for GSEA, the protein hits clustered in the heatmap (Fig. 2A) enriched several terms positively correlated with the MUT-KO condition, such as *mitochondrial membrane organization* and *cristae formation*, *organic acid transport*, *phospholipid* and *branched-chain amino acid (BCAA) metabolism*, and *fatty acyl-CoA* and *carbohydrate biosynthetic processes* (Fig. 2B, C). These terms correspond with several known features linked to MMA pathophysiology that affect the main energy biochemical pathways and mitochondrial routes within the cell, suggesting that the proteomics profile could be well associated with the disease. On the other hand, GSEA correlated other terms with MMA, such as the slowdown of *EGFR signaling* and *hormone metabolic process*, or the potentiation of *protein ubiquitination reactions* (Fig. 2B, C).

**Fig. 3:**
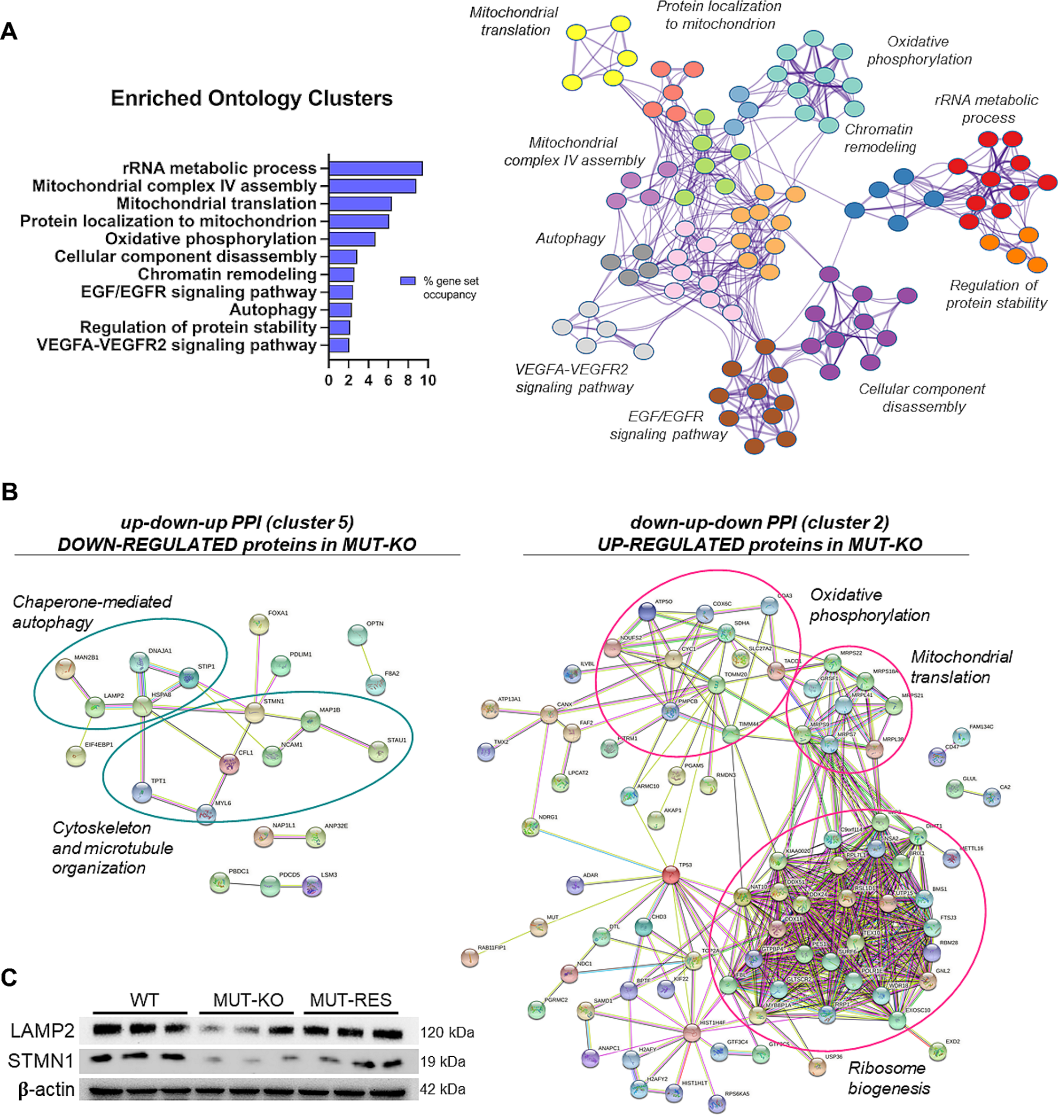
Functional enrichment analysis of the differential MUT-KO proteome. **(A)** The most significant (*p* < 0.01), non-redundant GO enriched clusters were selected in Metascape, and highlighted as global network with different colored nodes. In fact, a subset of representative GO terms was selected from a full cluster (according to q-values, percentage of gene set occupancy, and limiting redundancy) and converted into a network layout, where the color of the nodes represents the cluster identity. **(B)** STRING PPI networks enriched by the down-regulated proteins (cluster 5, Fig. 1) and up-regulated ones (cluster 2, Fig. 1), with details of the selected significant BP (FDR < 0.05). **(C)** Immunoblotting analysis of LAMP2 and STMN1 proteins in three biological replicates for HEK 293 WT, MUT-KO, and MUT-RES cells; β-actin was used as loading control

We further investigated the detailed pathways affected in MMA, inspecting the differential proteins in MUT-KO belonging to the clusters extracted from the statistical analysis of DIA proteomics data. Of particular interest were the clusters 2 and 5 (Fig. 1A, C), which showed the most sharply defined quantitative trends from negative–to positive–to negative values, and vice versa, in the order WT–MUT-KO–MUT-RES. Excluding MUT protein, cluster 5 included 44 proteins with the up-down-up trend, while cluster 2 included 102 proteins with the down-up-down trend (Supplementary Table 1). The total of 146 differential proteins (clusters 2 and 5) were analyzed in Metascape for the enrichment of ontology clusters, showing MMA-associated dysfunctions such as mitochondrial routes, as well as defects in *autophagy*, *RNA metabolism*, or *protein stability* (Fig. 3A). Then, two protein-protein interaction networks (PPI) were built for the up-down-up (cluster 5) and down-up-down (cluster 2) protein groups, separately (Fig. 3B). The PPI of down-regulated proteins (cluster 5) enriched *cytoskeleton and microtubule organization* and *chaperone-mediated autophagy*. Two of the most interesting dysregulations not yet associated with MUT deficiency occurred for lysosome-associated membrane glycoprotein 2 (LAMP2) and stathmin (STMN1). Proteomics data were validated through the immunoblotting of LAMP2 and STMN1 both showing decreased detection signals in MUT-KO cells (Fig. 3C). On the other hand, the majority of up-regulated proteins (cluster 2) in MUT-KO were involved in *oxidative phosphorylation*, *mitochondrial translation*, and *ribosome biogenesis* (Fig. 3B).

### The proteome of MMA fibroblasts highlights the alteration of MUT-related functions

To confirm alterations in discovered pathways or generate hypotheses about novel adaptive mechanisms in MMA, we adopted primary dermal fibroblasts of two MMA patients both diagnosed as mut0 (named M01 and M02), and HDFa fibroblasts as control (CTRL) for a shotgun label-free quantitative (LFQ) proteomics experiment. All the conditions (M01, M02, CTRL) were grown as four independent biological replicates and analyzed by LC-MS/MS (total *n* = 12). The global proteomic dataset contained 3101 proteins that were correctly identified with a minimum of one unique peptide in at least 30% of the 12 LC-MS/MS runs (this threshold was set to retain in the dataset the MUT protein of CTRL cells).

**Fig. 4:**
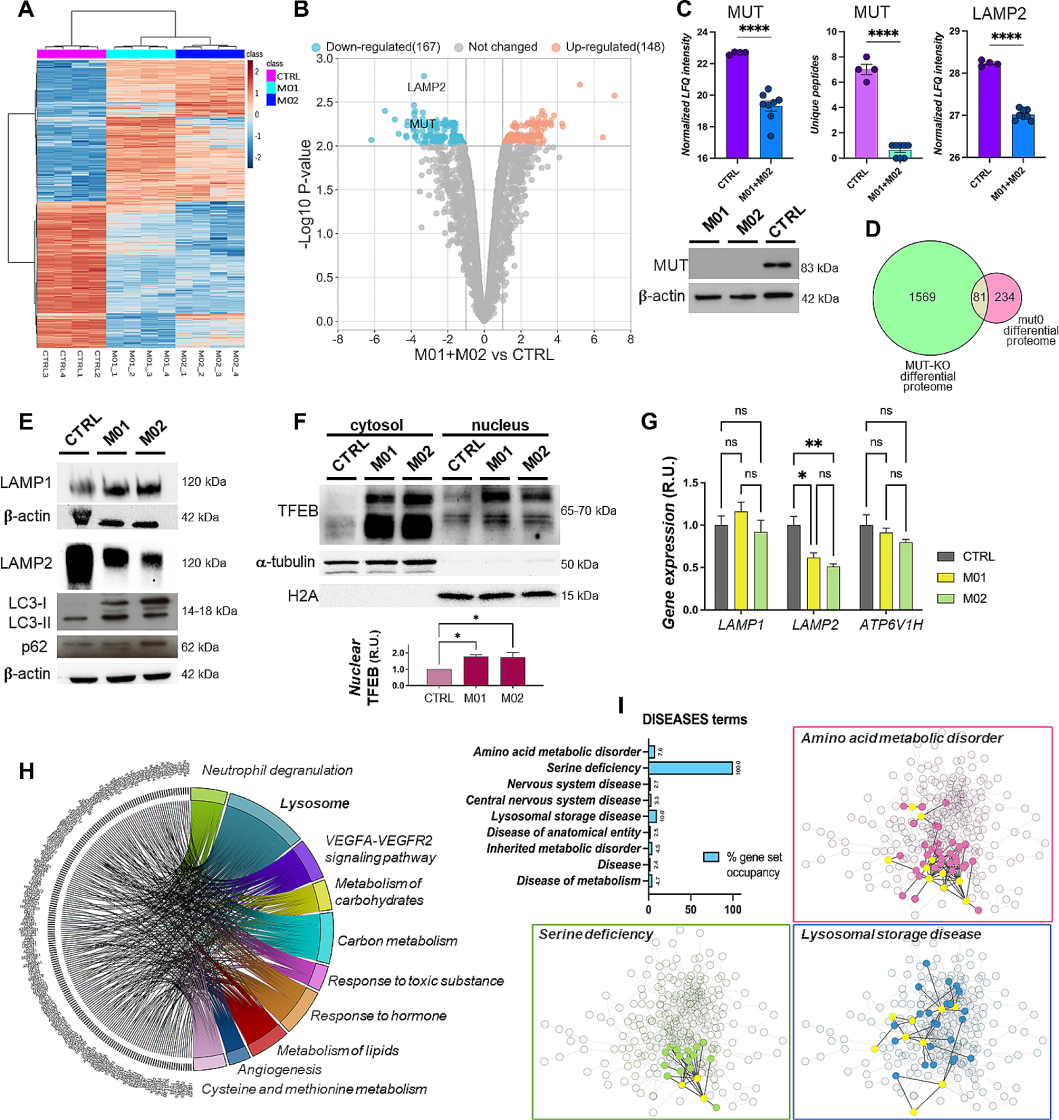
Functional characterization of the proteome of mut0 patients’ fibroblasts. **(A)** Heat map and hierarchical clustering reporting LFQ intensities of the proteins identified and the relations between the CTRL, M01, and M02 datasets. **(B)** Volcano plot analysis of significantly regulated proteins (–log10 p-value > 2) in the comparison M01 + M02 versus CTRL, with details of MUT and LAMP2 proteins. **(C)** Bar plot showing the statistical difference of LFQ intensities of the replicates of M01 + M02 versus CTRL for MUT and LAMP2 (upper panels). An additional plot was reported for the number of MUT’s unique peptides identified by LFQ proteomics in the analyzed conditions (upper panels). The statistical significance (****p < 0.0001) was calculated by unpaired t-test. WB analysis of MUT protein was performed on M01 and M02 patients’ cells; β-actin was used as loading control (lower panel). **(D)** Venn diagram reports the overlap between MUT-KO and mut0 differential proteomes. **(E)** Representative WB of lysosomal (LAMP1, LAMP2) and autophagy-related (LC3, p62) proteins. For all the tested proteins, β-actin was used as loading control. **(F)** Representative WB of cytosolic and nuclear TFEB levels; α-tubulin and histone H2A were used as cytosolic and nuclear loading controls, respectively. TFEB nuclear levels were measured by densitometry, normalized with H2A signals, and reported as relative units (R.U.) (mean ± SEM) values using the value of CTRL as unit (right panel). Statistical analysis was performed by one-way ANOVA; *p < 0.05. **(G)** Gene expression levels of LAMP1, LAMP2 and ATP6V1H were measured by RT-qPCR and calculated with the 2^−∆∆Ct^ method, using *β-actin* as reference gene for normalization. Mean values of three independent experiments were reported as R.U. (mean ± SEM). Statistical significance was calculated by two-way ANOVA; **p < 0.01, *p < 0.05, ns = not significant. **(H)** Chord plot displaying the relationships between the differential proteins and the terms enriched by Metascape analysis in mut0 fibroblasts. **(I)** Enrichment of the Diseases terms category obtained through the STRING app in Cytoscape. The terms were plotted according to their % of gene set occupancy, with a detail of the PPI network of the proteins belonging to the most enriched terms with their first neighbors

Protein distribution in the heatmap clustering showed a fair overlay of the selected features in M01 and M02, with only a few exceptions (Fig. 4A). Thus, the high reproducibility of quantitative data permitted to analyze the M01 and M02 fibroblasts’ proteomes together (M01 + M02) for the statistical comparison against the CTRL proteome. Therefore, volcano plot analysis revealed that 315 proteins were differentially regulated (FDR = 0.01), with 167 being less abundant and 148 more abundant in mut0-fibroblasts (Fig. 4B and Supplementary Table 2). As a comparison with the MUT-KO proteome, we recovered MUT and LAMP2 among the down-regulated proteins, besides many others (Figs. 3C and 4B). In fact, the significance of the statistical difference of the normalized LFQ intensities between M01 + M02 and CTRL was elevated (*p* < 0.0001) for both MUT and LAMP2 (Fig. 4C). In addition, only one unique peptide of MUT was found in a few M01 or M02 samples compared to CTRL (average of 7 peptides) (Fig. 4C). Indeed, in accordance with MS data, no MUT-antibody signals appeared in M01 and M02 cells (Fig. 4C, lower panel). Globally, the comparison of MUT-KO and mut0 differential proteomes resulted in 81 common species (Fig. 4D and Supplementary Table 3).

We also confirmed by western blot (WB) the reduction of LAMP2 levels in both M01 and M02 fibroblasts compared to CTRL (Fig. 4E). LAMP2 is a component of the lysosomal membrane and its deficiency causes a lysosomal storage disease (LSD) known as Danon disease associated with autophagy deregulation [19]. Hence, we investigated possible changes in the levels of other lysosomal and autophagy markers in mut0 fibroblasts, finding LAMP1 highly increased (Fig. 4E). Moreover, the levels of LC3 and its cleaved form LC3-II [20, 21] were higher in M01 and M02, whereas a slight increase of p62 was observed (Fig. 4E). To assess whether this landscape was caused by an induction of autophagy, we evaluated the levels of transcription factor EB (TFEB), which appeared strongly up-regulated in MMA cells. In particular, we separated the cytosolic and nuclear fractions of TFEB (both higher than CTRL) and focused on nuclear TFEB to provide indications of TFEB activity (Fig. 4F, quantitative plot shown). Given the higher levels of nuclear TFEB in MMA cells, we expected that transactivation ability of the factor was increased as well, possibly resulting in a higher expression of its target genes. Hence, we tested by RT-qPCR the expression of two known TFEB target genes, namely *LAMP1* and *ATP6V1H*, whose transcription is particularly sensible to TFEB [22–24]. Surprisingly, these two genes showed no change in their transcript levels (Fig. 4G). This finding suggests that, despite the higher levels of nuclear TFEB in mut0 cells, the transactivity of this factor is not increased and this, in turn, may indicate no rise in autophagy induction in MMA cells. Moreover, given the discrepancy between transcript and protein levels of LAMP1, we also measured *LAMP2* gene expression. Opposite to *LAMP1*, *LAMP2* transcript strongly diminished in MMA (Fig. 4G) as per the protein. Our data indicate that while LAMP2 down-regulation in MMA can be due to transcriptional routes, a post-transcriptional mechanism may regulate LAMP1 protein levels.

Nonetheless, functional enrichment of ontologies connected with MMA proteome highlighted *lysosome* as the most crowded term (Fig. 4H). Other significant terms, including *VEGFA-VEGFR2 signaling pathway*, *metabolism of carbohydrates*, *response to hormone*, and *metabolism of lipids* were identified also in MUT-KO proteome (Figs. 2 and 3A). In addition, the *cysteine and methionine metabolism* term was found enriched in another study reporting the dysregulation of serine metabolism in patients’ fibroblasts as a novel area of cellular dysfunction for MMA [25]. In fact, when we investigated the association of the mut0 differential proteins with the clinical features possibly associated with MMA, we identified *serine deficiency* with the highest percentage of gene set occupancy (100%) (Fig. 4I). *Amino acid metabolic disorder* and *Lysosomal storage disease* terms accounted for 7.6% and 10%, respectively. While the alteration of amino acid metabolism is a known feature of MMA, the latter association with LSD would represent a novel characteristic of the disease, thereby creating a new link between the organic acidemias and the lysosomal dysfunctions.

### Lysosome morphology is altered in MMA

**Fig. 5:**
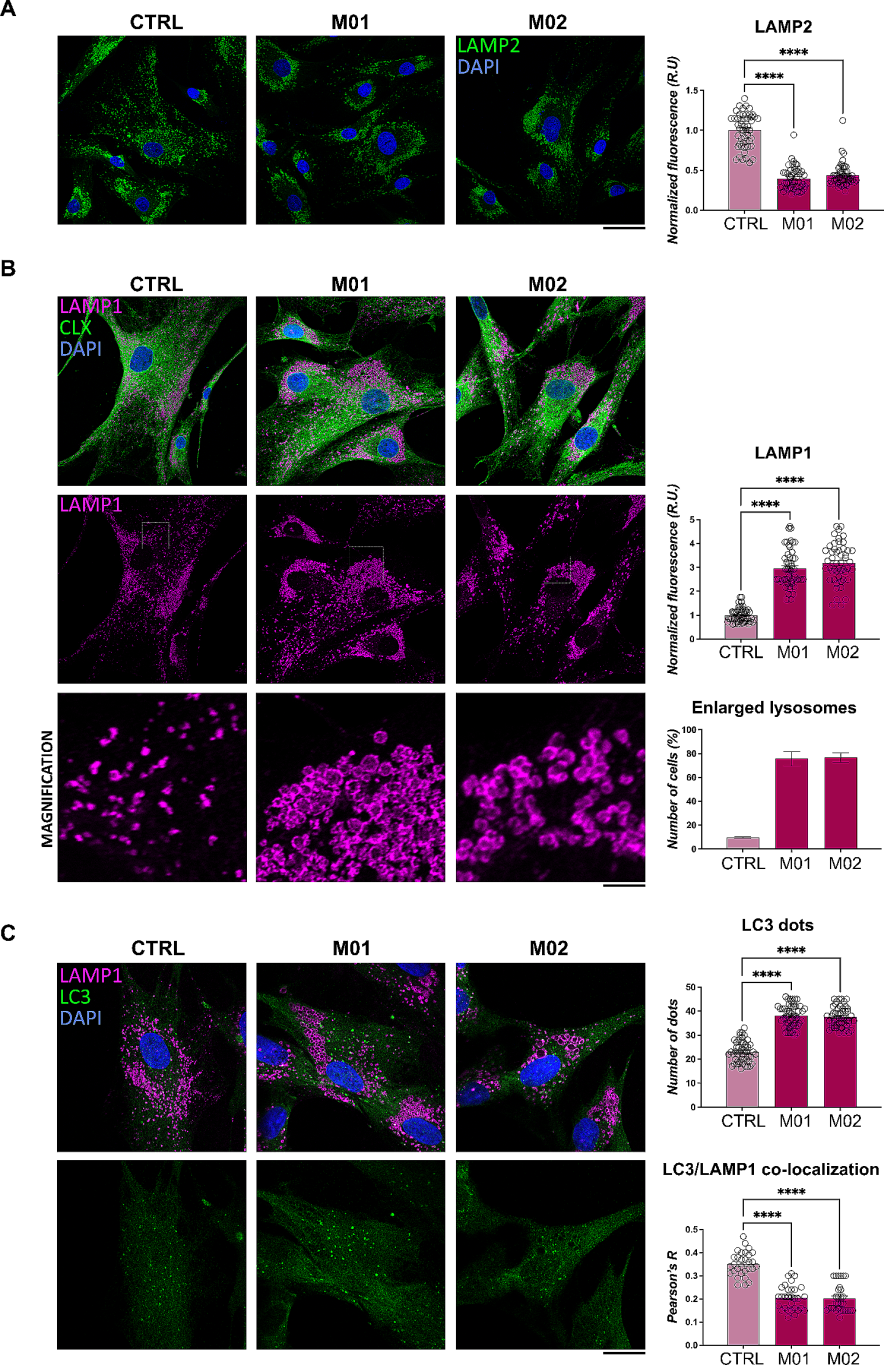
Structural alterations of lysosomes in MUT-deficient fibroblasts from MMA patients. **(A)** Images of cells immuno-stained with anti-LAMP2 antibody quantifying the mean fluorescence intensity per cell (right panel) reported as mean ± SEM. **(B)** Images of cells immuno-stained with anti-LAMP1 and anti-CLX antibody. LAMP1 mean fluorescence intensity per cell was also measured (higher-right panel) and mean ± SEM is shown. The percentage of cells with enlarged LAMP1-positive structures (lysosomes) was also calculated (lower-right panel) and mean ± SEM is shown. **(C)** Images of cells immuno-stained with anti-LAMP1 and anti-LC3 antibody; number of LC3 dots per cell were also measured (left-upper panel); mean values ± SEM are shown; the percentage of cells with enlarged lysosomes was also calculated (left-lower panel); co-localization of LC3 and LAMP1 was also measured by Pearson’s correlation of the signals and reported as Pearson’s R coefficient values (left-lower panel). For all microscopy images the scale bar is 20 μm. For all the comparisons in the figure, statistical analysis was performed by one-way ANOVA; ****p < 0.0001

The hints obtained by previous analyses led us to investigate a possible compromission of the lysosomal and autophagic pathway in MMA. In particular, these observations suggested that the autophagy machinery could be compromised in MMA following LAMP2 down-regulation. Indeed, a significant reduction of LAMP2 was further validated by immunofluorescence and confocal microscopy analyses (quantitative plot shown) (Fig. 5A). Nonetheless, confocal microscopy was used to analyze lysosomal morphology and distribution. In particular, we adopted a protocol which allowed us to analyze the lysosomal morphology by preserving lysosome integrity. A co-staining of LAMP1 lysosomal marker and calnexin (CLX) as a marker of the endoplasmic reticulum (Fig. 5B) would have highlighted a possible endoplasmic reticulum stress, being this latter hypothesis in accordance with proteomics data obtained in MUT-KO cells that showed an up-regulation of a large protein network related with ribosome biogenesis (Fig. 3B). Although no significant differences were noticed in CLX staining between mut0 and control fibroblasts (quantitative plot not shown), on the other hand, the fluorescence signals of LAMP1 were more represented in MMA cells (quantitative plot shown) (Fig. 5B), confirming WB results (Fig. 4E). Interestingly, LAMP1 staining showed the presence of enlarged lysosomes in both M01 and M02 fibroblasts (Fig. 5B). Moreover, M01 and M02 displayed a centrosomal localization of the enlarged lysosomes (Fig. 5B), a hallmark of many LSD [26, 27].

Furthermore, a co-staining with anti-LC3 and anti-LAMP1 antibodies showed a significant increase in the number of LC3-positive structures (LC3 dots) and a decrease of LC3/LAMP1 co-localization in mut0 cells (Fig. 5C). This aspect led us to hypothesize that such morphologically abnormal lysosomes could be also dysfunctional, thus accumulating undegraded material in the lumen and being trapped in the perinuclear region of the cells. While the increase of LC3 would suggest a positive regulation of autophagosome biogenesis, the decreased co-localization of LC3/LAMP1 advocates a reduced fusion of autophagosomes with lysosomes.

### Lysosome functionality is altered in MMA

These aspects led us to hypothesize that such morphologically abnormal lysosomes could be also dysfunctional, thus accumulating undegraded material in the lumen and being trapped in the perinuclear region of the cells. While the increase of LC3 would suggest a positive regulation of autophagosome biogenesis, the decreased co-localization of LC3/LAMP1 advocates a reduced fusion of autophagosomes with lysosomes. To further shape this view, we measured the autophagic flux of mut0 and CTRL cells by time-course treatment with Bafilomycin A1 (Baf A1) [28, 29].

**Fig. 6:**
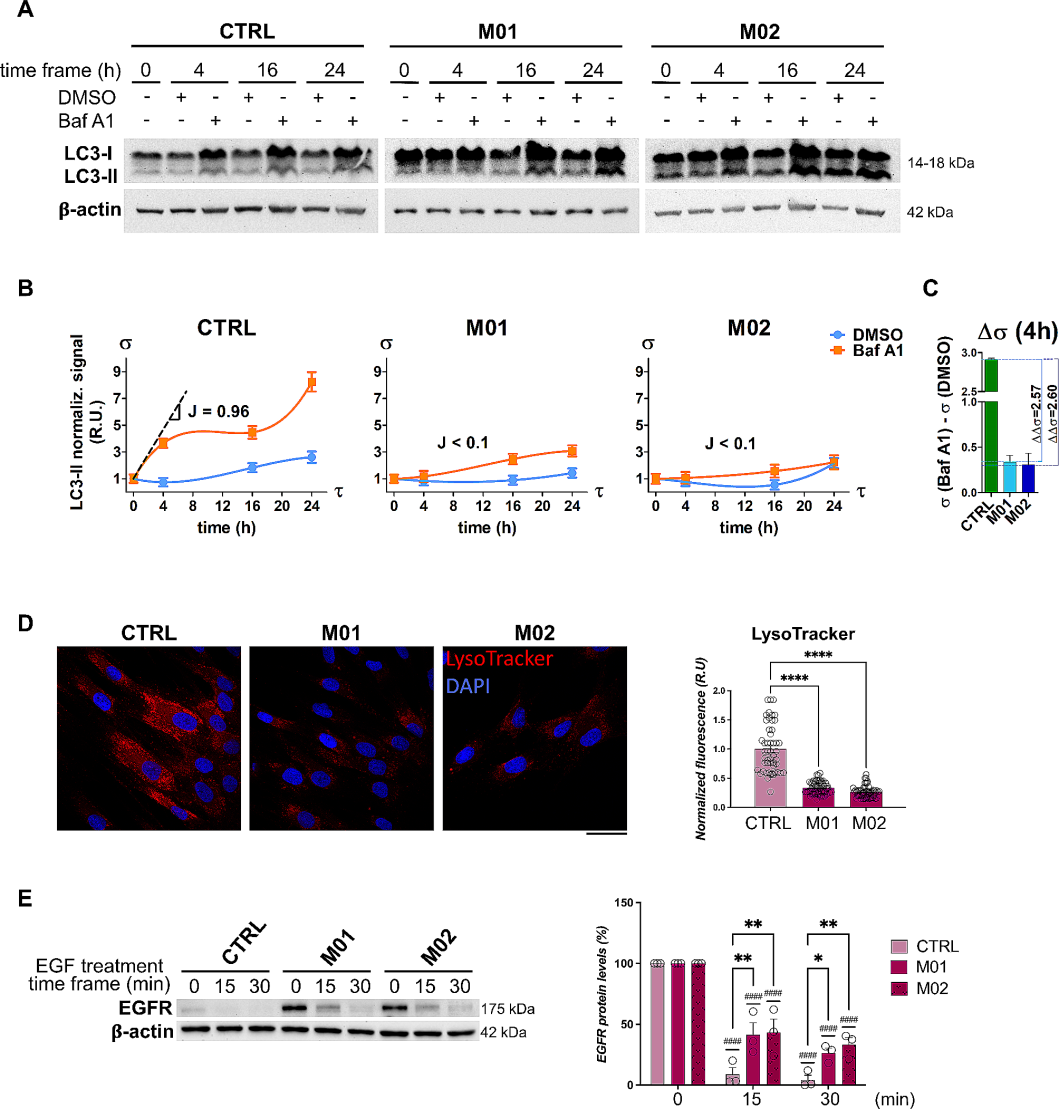
Lysosomal and autophagic dysfunctions in MUT-deficient fibroblasts from MMA patients. **(A)** The autophagic flux was measured in CTRL, M01 and M02 cells quantifying by WB the levels of LC3-II protein after treatment with Bafilomycin A1 in a time-course experiment (4, 16, 24 h). The β-actin was used as loading control. **(B)** Normalized LC3-II signals (σ) were used to draw regression curves that were used to mathematically calculate the autophagic flux (J). The data on the graph are the average ± SEM of two independent WB replicates. **(C)** For each of the treated cell lines (CTRL, M01 and M02) we calculated the Δσ as difference of the treatment at 4 h, i.e. σ(Baf A1) – σ(DMSO). In addition, the ΔΔσ was calculated for MMA cells as differences of M01 and M02 Δσ with the CTRL Δσ, respectively. **(D)** Images of cells stained with LysoTracker. The fluorescence intensity per cell (right panel) was reported as mean ± SEM. Statistical analysis was performed by one-way ANOVA; ****p < 0.0001. **(E)** EGFR degradation assay consisted of WB analysis of EGFR in cells starved and stimulated with EGF (15, 30 min); EGFR protein levels were measured by densitometry, normalized with β-actin signals, and reported as percentage values (mean ± SEM) using the value at time 0 (starved but not stimulated cells) as 100% reference (right panel). Statistical analysis was performed by two-way ANOVA for the comparisons M01 and M02 vs. CTRL at 15 and 30 min of EGF stimulation (*p < 0.05, **p < 0.01) and at 15 min vs. 0 min, and 30 min vs. 0 min within each cell line (####*p* < 0.0001)

In particular, we observed that the accumulation of LC3-II (σ) was enormously enhanced upon Baf A1 treatment in CTRL fibroblasts (Fig. 6A) for which we calculated an autophagic flux (J) of 0.96 (Fig. 6B). On the other hand, Baf A1-treated mut0 cells showed only a small difference in the levels of LC3-II if compared with their untreated counterparts (Fig. 6A), reporting values for J < 0.1 for both M01 and M02 (Fig. 6B). Comparing the levels of Δσ in MMA cells after four-hour autophagy inhibition, M01 and M02 showed ΔΔσ values of 2.57 and 2.60 with respect to CTRL (Fig. 6C). Such evidence demonstrates that the autophagic flux in MMA is significantly lower than CTRL, not being particularly affected by Baf A1.

Reflecting on the above-described structural and functional alterations, we further confirmed that MMA lysosomes do not work properly. In our previous reports, we showed that MUT-deficient cells do not suffer a significant decrease of NR – or MTT – signals in standard culture conditions probably due to metabolic adaptation, unless exposed to propionate overload to force the propionate pathway [18]. Indeed, NR uptake assay to test lysosome functionality and MTT cell viability assay in mut0 fibroblasts without propionate overload did not show significant differences with CTRL, as expected (Fig. S1).

However, LysoTracker staining confirmed impaired activity of MMA lysosomes, showing significantly lower signals in M01 and M02 fibroblasts than controls (quantitative plot shown) (Fig. 6D). These observations imply a reduced acidity (i.e., pH increase) of lysosomes with a possible compromission of at least some digestive functions of these organelles.

Therefore, to test the degradation capability of the lysosomal compartment, we analyzed the degradation dynamics of the epidermal growth factor receptor (EGFR) upon stimulation with its extracellular ligand EGF. Notably, once bound by EGF, the EGFR is internalized by clathrin-mediated endocytosis and delivered to the lysosomal compartment for its degradation [30]. A lower kinetic of EGFR degradation could support the compromission of lysosomal functionality [30]. Hence, we analyzed EGFR protein levels by WB and compared fibroblasts treated with EGF for two different time frames (i.e. 15 min and 30 min) with untreated fibroblasts (i.e. 0 min) (Fig. 6E). We observed that the percentages of residual EGFR protein 15 min and 30 min after EGF treatment were significantly higher in mut0 samples (Fig. 6E). Therefore, these data strongly support the above-mentioned hypothesis on the impaired lysosomal degradative activity and functionality in MMA fibroblasts cells.

### Morphology and function of MMA-lysosomes are rescued upon DMBA treatment

Finally, we attempted to understand whether lysosomal alterations could be directly connected with MUT and the subsequent accumulation of propionyl-CoA metabolites. To this aim, we used the 2,2-dimethylbutanoic acid (DMBA) as a drug for treatment of mut0 cells. Actually, DMBA – also named HST5040 – is a low-molecular-weight carboxylic acid that can readily form CoA esters, thus modifying the distribution of the cellular acyl-CoA pools and being, in turn, effective in reducing abnormally elevated propionyl-CoA and methylmalonyl-CoA levels in cells and tissues [31].

**Fig. 7:**
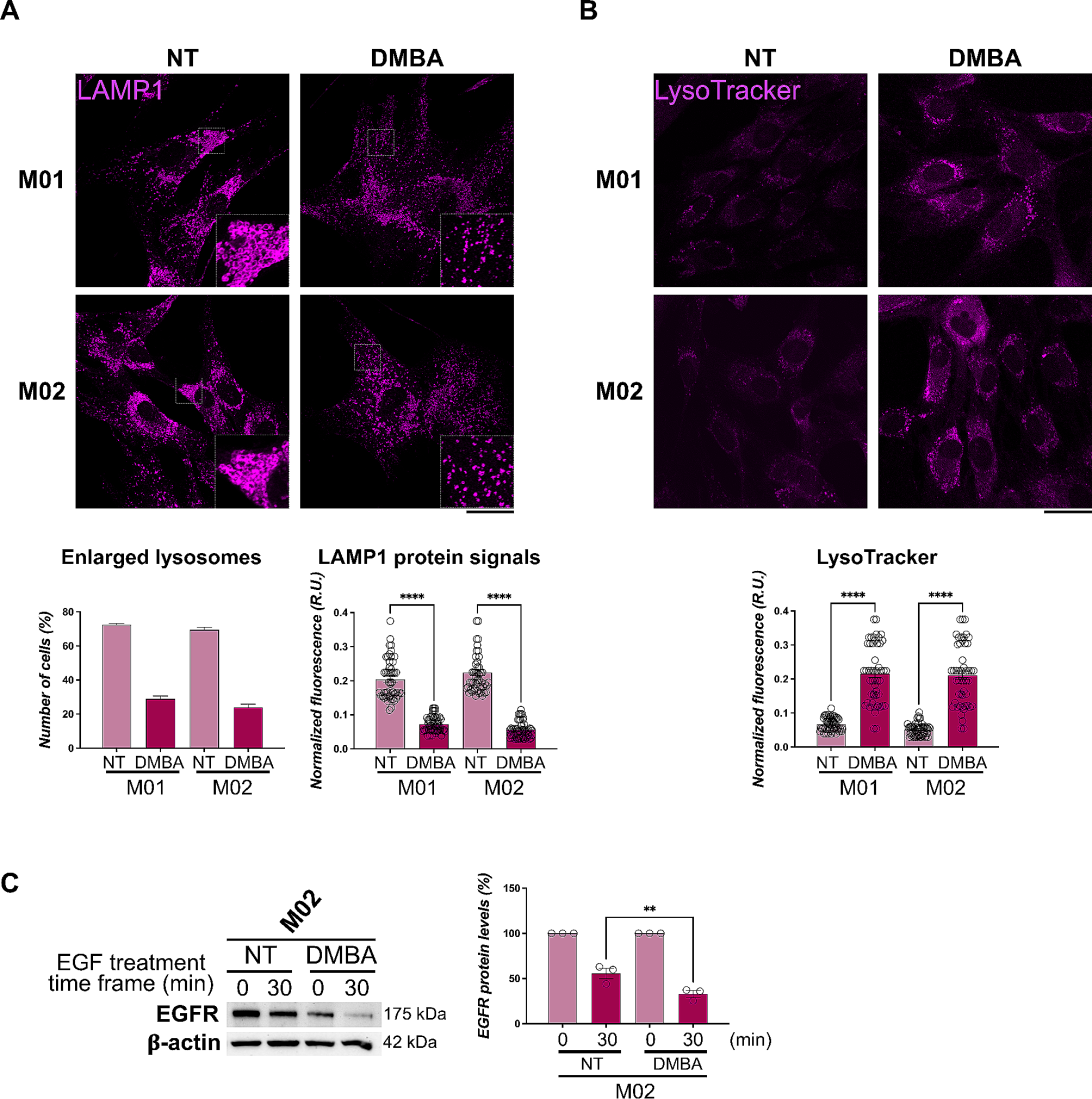
Structural and functional alterations of MMA lysosomes are rescued upon DMBA treatment. **(A)** Images of LAMP1 immuno-staining in M01 and M02 cells treated with DMBA and non-treated (NT) cells; the percentage of cells with enlarged LAMP1-positive structures (lysosomes) was reported as mean ± SEM (lower panel). **(B)** Images of LysoTracker staining of NT and DMBA-treated M01 and M02 cells. The fluorescence intensity per cell (lower panel) was reported as mean ± SEM. Statistical analysis was performed by one-way ANOVA; ****p < 0.0001. **(C)** EGFR degradation assay with DMBA treatment and densitometry analysis (right panel) were performed in M02 cells as reported in Fig. 6E; **p < 0.01. All the WB and microscopy images are representative of at least three independent experiments. For all microscopy images the scale bar is 20 μm

Therefore, we wondered whether DMBA could revert the lysosomal morphologic alterations observed, demonstrating in the positive case the connection between MUT deficiency and acyl-conjugates accumulation with lysosomal impairment. We treated mut0 cells with DMBA and analyzed lysosomal distribution and morphology through LAMP1 staining. Comparing DMBA-treated fibroblasts with their untreated counterparts, we showed that the drug was able to significantly reduce the presence of abnormally enlarged lysosomes, thus rescuing the lysosomal morphology defects (Fig. 7A). CTRL cells treated with DMBA did not display any significant differences in lysosomal morphology compared with non-treated HDFa cells (data not shown). Besides rescuing the morphology of MMA lysosomes, we checked whether their functionality could be recovered as well. Indeed, DMBA treatment was able to restore the fluorescence signals of the LysoTracker probe in both M01 and M02 patient cells (Fig. 7B), demonstrating itself as a drug capable to recover the lysosome functionality clearly damaged upon MUT-deficiency and acylated-metabolites accumulation. As further evidence of the recovery of MMA-lysosomes function, DMBA treatment improved the digestive capabilities of these organelles enhancing the EGFR degradation. In fact, a reduced band for EGFR signal was observed in M02 samples after DMBA administration (Fig. 7C).

## Discussion

The correct functioning of lysosomes as degradative organelles is essential for maintaining cellular homeostasis; however, they have been found to participate in many other cellular processes, including plasma membrane repair, cell adhesion and migration, cell death, metabolic signaling, gene regulation, etc [32]. . . In recent years, lysosome dysfunction has been shown to underlie not only rare LSD but also other diseases [32]. Indeed, to the best of our knowledge this association with MMA has never been contemplated yet.

Here, we show the use of a multi-proteomic approach to disclose novel unknown pathological mechanisms connected with MMA employing different cellular models of MUT deficiency [18, 33, 34]. By combining high-resolution DIA proteomics and bioinformatic analyses, we highlighted the dysregulation of proteins and relative pathways associated with MMA pathophysiology with the principal affection of energy metabolism and mitochondrial paths. More interestingly, we found out and validated the lysosomal involvement as a novel feature of MMA, possibly involved in intracellular mechanisms of damage. Our analyses found alterations that in part confirm the results of a recent iTRAQ proteomics work performed on MMA patients’ serum that enriched KEGGs related to lysosome and cholesterol metabolism pathways, with no further investigation or exploration on patients’ samples [35].

In recent years, mitochondrial dysfunctions in MMA have been deeply studied, providing insights into the damaged processes that normally sustain metabolic and energetic homeostasis. Recently, it has been proven that the accumulation of non-functional mitochondria in MMA cells is dependent on impaired cellular clearance mechanisms or their exacerbation. In fact, whereas the autophagic machinery naturally impedes the accumulation of non-functional organelles [36–39], a defective mitophagy was observed in MUT-deficient cells [40]. Moreover, anomalies in the PINK1/Parkin mitophagy system would provoke the accumulation of dysfunctional mitochondria triggering, in turn, epithelial stress and cell damage [18, 40].

According to these findings, we add a piece of knowledge that could connect the impaired mitophagy with alterations of the lysosomal activities and structures, including autophagy and digestive functions. In particular, we confirmed that LAMP2 protein and its transcript are strongly down-regulated. On the other hand, LAMP1 showed an up-regulation at the protein level in MMA cells but such deregulation did not occur at transcriptional level. The discrepancy between the trend of abundance of LAMP1 and LAMP2 could be due to post-transcriptional mechanisms (e.g. accumulation of engulfed lysosomes) that upregulate LAMP1 levels, or to compensatory mechanisms that regard the relationship between these two lysosomal-associated membrane proteins. Hence, according to some studies, it would seem that the deficiency of LAMP-1 is accompanied by up-regulation of LAMP2 [41].

LAMP2 has a defined role in phagolysosome biogenesis, mediating the fusion of lysosomes with autophagosomes, an essential step for the effective completion of the autophagy [42]. As necessary parallelism, Danon disease (DD) is a LSD due to deficiency of LAMP2, and characterized by defective lysosomal function, accumulation of abnormal autophagic vacuoles in cells and autophagic impairment, presenting with multisystem disorders principally involving heart, skeletal muscle and central nervous system [43]. Indeed, it was reported that LAMP2-deficient cells from individuals (i.e. DD patients) or (genetic KO) mice have increased numbers of autophagosomes in vivo and in vitro [44]. For these reasons, autophagy-guided interventions have been proposed to modify the phenotype of DD. Autophagy induction in Danon mice exacerbates the disease phenotype fostering dilated cardiomyopathy. Autophagy inhibition better supports mice phenotype but is not totally curative [45]. Given the evidence that mitochondria dysfunction and fragmentation can result in Danon pathology, and MMA is characterized by similar damaging processes [6, 18, 25, 40, 46–48], here we show that diseases with different etiology may be united by the same pathological mechanisms. This is instrumental considering that MMA patients are also characterized by episodes of congenital heart disease, and dilated or hypertrophic cardiomyopathy depending on MMA subtypes [47].

On the other hand, further studies explained the involvement of LAMP2-deficiency in generating oxidative stress (ROS) through the reduction of cytosolic cysteine concentration, resulting in low glutathione (GSH), lower antioxidant capability and increased mitochondrial lipid peroxidation [49]. Accordingly, as seen in our previous surveys, dysfunctional mitochondria in MMA produce high amounts of ROS, with cells showing increased susceptibility to stress [18]. Such oxidative alterations can be strictly connected with LAMP2 down-regulation in MMA, whereas studies in LAMP2-deficient (KO) mice provided evidence for mitophagy impairment proving that LAMP2 is required for mitochondria clearance and turnover [50]. On the same basis, LAMP2 alterations co-occurred with increased p62 and LC3 content in LAMP2-KO mice [50]. In accordance with these findings, our results displayed higher levels of LC3 and p62 and LAMP1 in mut0 fibroblasts.

LC3 and p62 are crucial markers to monitor the autophagic flux by quantitative analysis (WB) or observing the formation and variations of autophagosomes/autolysosomes by immunofluorescence and microscopy, despite not providing a complete picture of such a dynamic process [51, 52]. The number of autophagosomes can increase when the autophagic flux is stimulated, as well as the content of LC3 that is recruited to the autophagosomal membranes [53] and of p62, which binds to LC3 [51]. On the other hand, in absence of autophagy enhancement, autophagosomes can accumulate for defective degradation [29, 54]. Actually, through our experimental evidence the autophagy landscape of MMA would suggest an increase of autophagosome intracellular pool that is not dependent upon autophagy induction. In fact, despite higher levels of nuclear TFEB in mut0 cells, the capability of this factor in transactivating the expression of target genes seems to be unaltered. Moreover, in accordance with the hypothesis of autophagosome accumulation in mut0 fibroblasts, bafilomycin A1 has very limited effect in these cells which show an autophagic flux more than 10-fold lower if compared with that of CTRL fibroblasts. These findings, in turn, suggest that the observed increase in autophagosome markers is likely due to autophagosome accumulation rather than autophagogenesis induction.

Accordingly, the morphological alterations of mut0 lysosomes presenting themselves as abnormally enlarged LAMP1-positive structures indicate a block of autophagosome-autolysosome fusion, explained by a reduced co-localization of LC3/LAMP1 [53, 55] and reduced LAMP2 levels, which is directly required for autophagosomes-autolysosomes fusion during autophagy [44]. In fact, cells undergoing autophagy should be characterized by the co-localization of p62, LC3, and other lysosomal markers [51]. Our results strongly prove that MUT-deficiency blocks the autophagosome-lysosome fusion and harms the autophagosomal clearance, resulting in accumulation of enlarged and engulfed lysosomes in the cytosol.

Furthermore, the direct connection between methylmalonyl-CoA mutase deficiency and lysosomal impairment is given with the evidence that mut0 cells treated with DMBA are able to rescue the lysosomal phenotype. In the last couple of years, DMBA has been considered a therapeutic candidate drug for propionic acidemia and MMA [31]. In fact, the ability of DMBA to drive the redistribution of free and conjugated CoA pools has been employed as a means to reduce aberrant levels of propionyl-CoA and methylmalonyl-CoA [56]. Surprisingly, owing to the net decrease of CoA metabolites, either by lowering the production or promoting their clearance [56], the percentage of enlarged lysosomes in mut0 cells was dramatically reduced.

Finally, here we found that morphological alterations in MMA are also accompanied by functional impairment of lysosomal activities. Besides an evident disruption of the autophagic machinery, the digestive functions of MMA lysosomes appear significantly compromised as revealed by the low signals of the LysoTracker probe, thus implying a reduced acidity for increased pH in these organelles [57, 58]. Also, it was recently proven that LAMP proteins are both required for the maintenance of a highly acidic lysosomal pH via inhibition of the cation channel TMEM175 [59]. Indeed, mut0 fibroblasts were not able to degrade the EGFR into the lysosomes after internalization by endocytosis, clearly confirm the presence of functionally aberrant lysosomes. However, as a combined effect of the drug treatment, DMBA was also able to rescue the functionality of MMA-lysosomes by restoring the LysoTracker signals (correcting the acidity of these organelles) and improving their degradation capability.

As our findings directly connect MUT deficiency with structural and functional alterations of lysosomes, we propose the consideration of autophagy/lysosome-targeting molecules or pathways as novel area of intervention for complementary approaches in MMA. Interestingly, given the novel link between MMA and lysosomal dysfunction, we offer food for thought on possible future investigations to correctly define cobalamin processing; in fact, the biosynthesis of MUT cofactor adenosylcobalamin firstly takes place into the lysosome [60]. Thus, we suggest to investigate new options to rescue the lysosomal function as a therapeutic strategy for application in MMA patients in combination with toxic metabolites-lowering therapies and other supporting approaches to sustain secondary unbalances incurring in energy and homeostatic processes.

## Conclusions

In summary, the current work employed cellular models and patients-derived cells to refine and uncover new pathologic mechanisms in MMA. Our results establish an impairment of the autophagic machinery in MMA. Importantly, we identify either structural or functional lysosomal aberrations, defining the molecular and biochemical actors involved in such a scenario using a combination of multi-proteomics, bioinformatics and classical biochemical assays. The current study not only improves our understanding of the pathogenetic mechanisms of MMA but also draws attention to novel possible therapeutic options that could include the modulation of lysosomal and autophagic targets.

## Materials and methods

### Cell cultures

HEK 293 cells carrying the knockout of *MUT* gene (MUT-KO) and those with the rescue of the MUT protein expression (MUT-RES) were obtained from HEK 293 wildtype (WT) cells as previously reported [18].

Primary dermal fibroblasts of MMA patients, obtained from the Telethon-Gaslini Biobank [61], belong to two distinct patients (that we named M01 and M02) which had been both diagnosed as mut0 (i.e. complete lack of MUT enzymatic activity). Commercially available primary healthy human dermal fibroblast HDFa purchased from Thermo Fisher Scientific (Waltham, MA, USA) were used as a control (CTRL) for the experiments involving M01 and M02 cells.

Cells were cultured with high glucose Dulbecco’s Modified Eagle Medium (DMEM) (EuroClone, Paington, UK) supplemented with a 10–15% fetal bovine serum (EuroClone), 4 mM L-Glutamine (Sigma-Aldrich, St. Louis, MO, USA), 1% of the penicillin-streptomycin solution (Sigma-Aldrich) at 37 °C in a 5% CO_2_ atmosphere.

### LC-MS/MS and DIA proteomics of HEK 293 cell models

HEK293 cells were prepared for data-independent acquisition (DIA) proteomics analysis following Filter-Aided Sample Preparation (FASP) proteolysis, peptide quantification by amino acid analysis (AAA), and high-pH reversed-phase HPLC fractionation as explained in detail in the Supplementary Materials.

Prior DIA-MS analysis, peptide fractions (pH 8.0) were resolubilized in 15 µL of 0.1% TFA containing 1.5 µL of diluted iRT standard and first analyzed in data-dependent acquisition (DDA) by nanoLC-MS/MS using an Ultimate 3000 nano RSLC system coupled to a Q Exactive HF mass spectrometer (both Thermo Scientific, Germany). Peptides were preconcentrated on a 100 μm×2 cm C18 trapping column for 5 min using 0.1% TFA with a flow rate of 20 µL/min followed by separation on a 75 μm×50 cm C18 main column (both Acclaim Pepmap nanoviper, Thermo Scientific) with a 120 min LC gradient ranging from 3 to 35% of B (84% ACN in 0.1% formic acid) at a flow rate of 250 nL/min. The Q Exactive HF operated in DDA mode and MS survey scans were acquired from 300 to 1500 m/z at a resolution of 60,000 using the polysiloxane ion at m/z 371.1012 as lock mass [62]. The 15 most intense ions were isolated with a 1.2 m/z window and fragmented by higher energy collisional dissociation (HCD) with a normalized collision energy (NCE) of 27%, taking into account a dynamic exclusion of 20 s. MS/MS were acquired at a resolution of 15,000. Automated gain control (AGC) target values and maximum fill times were set to 3 × 10^6^ and 120 ms for MS, and 5 × 10^4^ and 200 ms for MS/MS, respectively.

Directly after the DDA runs, peptides corresponding to 0.5 µg (based on AAA) of each sample containing 1.5 µL of diluted iRT standard were analyzed using the DIA method using the same LC-MS/MS set as above. Samples were analyzed in a randomized order to minimize systematic errors and check for any carryover, with dedicated wash blank runs in-between. Furthermore, prior starting DIA runs of real samples, a quick DIA run was performed (called “scouting method”) in order to (i) determine m/z scan range, (ii) assess the number of data points per peak, and (iii) calculate number of DIA segments [63]. Based on the information from the scouting measurement, the Q Exactive HF operated in DIA mode and MS survey scans were acquired from 300 to 1,201 m/z at a resolution of 60,000. The AGC target and maximum fill time values were set to 3 × 10^6^ and 20 ms, respectively. For DIA, the settings were: resolution 30,000; AGC target value 3 × 10^6^; isolation window 43.9 m/z; DIA segments 21, with each isolation width overlapping of 1.0 m/z; HCD NCE: 27% and the maximum fill time was set to auto. Both full MS and DIA data were acquired in profile mode.

### DIA-MS data analysis

DDA-MS raw data of high-pH fractions were processed with Proteome Discoverer (PD) 1.4 (Thermo Scientific) [34]. The .msf output file was uploaded in Spectronaut Pulsar software (version 12.0.20491.8.23937) for spectral library generation, and DIA runs (including the “scouting method”) were uploaded for DIA analysis of protein identification and quantification (Supplementary Materials).

Protein identifications with their respective abundance values (PG.Quantity) were exported from Spectronaut and imported into Perseus (version 1.6.15.0) for further data analysis [64]. Proteins were retained if they were identified by a minimum of one unique peptide in over the 50% of the LC-MS/MS runs. Log2-transformed abundances were imputed to remove NaN values (width = 0.5; downshift = 2.5). Significant proteins were selected using a multiple-sample test one-way ANOVA, with a permutation-based FDR of 0.05. Post-hoc analysis was performed using Tukey’s HSD test with FDR of 0.05 on the significant hits of the ANOVA test. Proteins of interest were selected according to their abundance trends in the analyzed conditions upon cluster selection from heatmap.

### Targeted proteomics analysis by parallel reaction monitoring (PRM)

To further validate the knockout of MUT gene in HEK 293 cells, 3 biological replicates of each cell line (namely WT, KO and RES) were analyzed by parallel reaction monitoring (PRM). The assay method was developed using Skyline software 64-bit version 4.2.0.19072 [65]. The peptides were selected based on (i) uniqueness, (ii) fully tryptic with no missed cleavage sites, and (iii) length of 8–25 amino acid residues to ensure reliable protein identification. Additionally, the PD output file obtained from the DDA analysis (see above) was used as the reference spectral library. For the targeted assay, 12 unique peptides/precursors of human methylmalonyl-CoA mutase, mitochondrial (UniProt accession: P22033) were exported as inclusion list from Skyline for unscheduled or scheduled targeted PRM analysis.

Tryptic peptides (∼ 2 µg) of the corresponding samples were measured using an Ultimate 3000 nano RSLC system coupled to a Q Exactive HF mass spectrometer. The LC method was identical as described above and the Q Exactive HF was operated in PRM mode without any MS1 scan event using the following settings: MS/MS scans were acquired in the Orbitrap at a resolution of 30,000 with an AGC target value of 1 × 10^5^ ions and a maximum injection time of 100 ms. Precursor ions were isolated using 0.4 m/z width, and fragmentation was performed in the HCD cell with a NCE of 27% as triggered by the provided inclusion list. Thus, generated raw MS/MS data were imported into Skyline for visualization, manual validation, mass error (Δ ppm), and assignment of retention time boundaries (as predicted by Skyline).

### LC-MS/MS and LFQ proteomics of mut0 fibroblasts

Fibroblast samples were cultured (1 × 10^6^ cells; *n* = 4 per condition), lysed and subjected to on-filter proteolysis through S-Trap™ micro spin columns (Protifi, Huntington, WV, USA) as published [37, 66].

LC–MS/MS system consisted of an Orbitrap Exploris 240 mass spectrometer equipped with a Vanquish Neo UHPLC (both Thermo Fisher Scientific). The LC separation was carried out on a commercial 15 cm Acclaim PepMap 100 C18 column (1 mm i.d.×150 mm, Thermo Fisher Scientific) at a flow rate of 0.250 µL/min. The buffer A used for separation was 0.1% (v/v) formic acid in water, the buffer B was 0.1% (v/v) formic acid in 80% acetonitrile. Peptides were separated with a 77 min gradient as follows: 2% buffer B for 3 min, 30% buffer B from 3 to 63 min, 50% buffer B from 63 to 68 min, 90% buffer B from 68 to 73 min, 95% buffer B from 73 to 77 min for column wash and equilibration. Full MS scans were acquired from m/z 300 to 1800 with an Orbitrap mass resolution of 120,000. MS/MS scans were performed in DDA with top 20 scans mode. Tandem MS/MS were acquired with an Orbitrap mass resolution of 15,000 and using an isolation window of 2 Da. HCD fragmentation was set with a NCE of 30%. The dynamic exclusion time was set as 25 s.

MaxLFQ algorithm integrated within MaxQuant (v1.6.17.0) was used for the label-free quantification (LFQ) analysis of all the raw data. The human proteome database from UniProt (UP000005640, 82,678 entries, 2023) was used as reference proteome for the Andromeda search. FDR based on posterior error probability (PEP) was determined by searching a reverse database and was set to 0.01 for proteins and peptides. Statistical analysis and data visualization were performed using the Perseus software (version 1.6.15.0). Common contaminants, peptides only identified by site modification and reverse peptides were excluded prior further analysis.

### Bioinformatics analyses and data visualization

Several bioinformatics tools have been employed for univariate and multivariate statistical analysis, functional enrichment purposes, or data visualization.

GSEA v4.3.2 application was used for the Gene Set Enrichment Analysis (GSEA) of HEK 293 cells to find statistically significant differences between MUT-KO and WT + RES samples, enriching GO and Reactome terms [67]. Metascape was employed to highlight the network of HEK 293 differentially regulated proteins (both up and down) in MUT-KO and to enrich ontology clusters associated to them [68]. PPI networks of differentially abundant proteins and GO enrichment in HEK 293 or mut0 fibroblast cells were built using STRING v11.5 or the STRING app in Cytoscape v3.9.1 [69]. MetaboAnalyst 5.0 and SRplot software were used for data visualization (e.g. heatmap, PCA, volcano and chord plots) [64, 66, 70]. Finally, GraphPad Prism 9.0 was employed for statistical analyses and graphical representations.

### Western blot

All protein samples analyzed by Western blot (WB) in this paper underwent the same procedure. Cell pellets were lysed and protein extracts were fractionated by SDS-PAGE, and transferred onto nitrocellulose membranes using a Trans-Blot Turbo Transfer System (Bio-Rad, Hercules, CA, USA). For nucleus-cytosol subcellular fractionation, cytosolic and nuclear extracts were obtained using Qproteome nuclear protein kit (Qiagen Italia, Milan, Italy) [71]. Membranes were blocked for 10 min at RT with EveryBlot Blocking Buffer (Bio-Rad Laboratories). Each primary antibody (Supplementary Materials) was incubated O/N at 4 °C in EveryBlot Blocking Buffer. Immunoblot detections were carried out using horseradish peroxidase-conjugated antibodies (GE Healthcare, Piscataway, NJ, USA) and enhanced chemiluminescence (GE Healthcare, Piscataway, NJ, USA). Signals were visualized either with ChemiDoc Imaging System (Bio-Rad Laboratories) or X-ray film exposure. Each experiment was averagely performed in three biological independent replicates, whereas one representative image is shown.

### RT-qPCR

Reverse transcription (RT) real-time PCR was performed as previously reported [18] and detailed in Supplementary Materials.

### Neutral-red uptake, MTT and crystal violet assays

Neutral-Red (NR) uptake, MTT, and Crystal Violet assays were performed as previously reported [48, 72, 73] and detailed in Supplementary Materials.

### Fluorescence microscopy

Sterilized 12 mm-diameter round coverslips were placed into 12-well plates, and 4 × 10^4^ dermal fibroblasts were seeded into each well and incubated in standard culture conditions. After 48 h, the cells were washed and properly fixed for microscopy. For LC3/LAMP1 co-staining, the fixative was ice-cold 100% CH_3_OH, and the cells were incubated at 0 °C for 5 min. For all the other immuno-stainings, the fixative was paraformaldehyde (PFA) solution 4% in PBS (Santa Cruz Biotechnology), and cells were incubated at RT for 10 min. For LysoTracker staining, the culture medium was added with 50 nM LysoTracker® Red DND-99 (L7528, Thermo Fisher Scientific) and cells were incubated for 2 h before fixation. In all the cases, cells were washed three times in PBS after incubation in fixative.

Immunofluorescence staining was performed as previously reported [74, 75]. Antibodies used are reported in Supplementary Materials. Images were collected using a laser-scanning microscope (LSM 700, Carl Zeiss Microimaging, Inc., Jena, Germany) equipped with a Plan Apo 63X oil immersion (NA 1.4) objective lens. Statistical significance was calculated using one-way ANOVA (with Dunnet correction in the cases of multiple comparisons). LC3/LAMP1 co-localization was evaluated by Pearson’s correlation between the two signals and Person’s R coefficient was reported.

### Autophagic flux measurement

Either Bafilomycin A1 (Baf A1) (s1413, Selleckchem, Houston, TX, USA) or DMSO (for untreated controls) were added to cell cultures 24 h after seeding. For 0 h time point, cells received neither Baf A1 nor DMSO. Baf A1 solubilized in DMSO was used at a final concentration of 100 nM as elsewhere reported [28], while the same volume of DMSO was used for untreated controls. These experiments were performed in two independent replicates.

LC3-II signals were normalized with β-actin (σ), expressed as relative units with respect to the corresponding 0 h time point, and reported in the graph as average ± SEM. The regression curves were third-degree polynomial, and those obtained for Baf A1-treated samples were used to calculate the autophagic flux (J), similarly to as elsewhere reported [29].

Briefly, from the equation of the curve σ as a function of the time (τ), the first derivative ∂σ/∂τ was calculated and in turn used to determine the initial slope of the σ curve which consisted in the autophagic flux J.

### EGFR degradation assay

EGFR degradation assay was performed on cultured dermal fibroblasts as elsewhere reported [30]. Briefly, after 24 h the culture medium was removed (cells were washed twice with PBS) and replaced with DMEM without serum or nutrients in order to induce cell starvation [37]. After 24 h of starvation, the culture medium was replaced with fresh serum-free medium containing 100 ng/mL EGF (Thermo Fisher Scientific). Cells were incubated for two different time frames (15 and 30 min) before WB. Starved cells not stimulated with EGF (time 0) were used as a control. EGFR protein levels were measured by band densitometry using ImageJ software, normalized with β-actin signals and reported as percentage values using the value at time 0 (starved but not stimulated cells) as 100% reference. The experiment was performed in three independent replicates and mean values ± SEM were reported. Statistical significance was calculated by one- or two-way ANOVA.

### 2,2-dimethylbutanoic acid (DMBA) treatment

Dermal fibroblasts were treated with 2,2-dimethylbutanoic acid (DMBA, D152609 Sigma-Aldrich) after 24 h of culture, using fresh medium containing 100 µM of DMBA and kept in the cell incubator for 5 h. This concentration was used as reported [31] and according to viability assays (Fig. S2). Untreated cells incubated in fresh medium without DMBA were used as a control. DMBA-treated and untreated cells were analyzed by immunofluorescence with anti-LAMP1 antibody and with LysoTracker reagent as explained above. For EGFR assay, after 5 h of incubation with DMBA, 100 ng/mL EGF (final concentration) were added and cells were incubated for 30 min before to be harvested and analyzed by WB. Starved cells not stimulated with EGF (time 0) were used as a control. EGFR protein levels were measured, normalized and graphically reported as specified above. All the experiment were performed in three independent replicates and mean values ± SEM were reported. Statistical significance was calculated by one- or two-way ANOVA.

### Electronic supplementary material

Below is the link to the electronic supplementary material.

Supplementary Material 1

Supplementary Material 2

Supplementary Material 3

Supplementary Material 4

Supplementary Material 5

## Data Availability

The data that support the findings in this study are available in the manuscript and Supplementary Materials of this article. The MS data related to DIA and PRM proteomics have been deposited to the ProteomeXchange Consortium via the PRIDE partner repository [76, 77] with the dataset identifier PXD044101. The MS data related to LFQ proteomics have been deposited to the ProteomeXchange Consortium via the PRIDE partner repository with the dataset identifier PXD044025.

## Abbreviations

Baf A1: Bafilomycin A1
DDA: Data-dependent acquisition
DIA: Data-independent acquisition
DMBA: 2,2-dimethylbutanoic acid
EGFR: Epidermal Growth Factor Receptor
GSEA: Gene Set Enrichment Analysis
HDFa: Human dermal fibroblast adult
LAMP1: Lysosome-associated membrane glycoprotein 1
LAMP2: Lysosome-associated membrane glycoprotein 2
LFQ: Label-free quantification
LSD: Lysosomal storage disease
MMA: Methylmalonic acidemia
MUT: Methylmalonyl-CoA mutase
NR: Neutral-Red
PRM: Parallel reaction monitoring
WB: Western blot
