# Supplementary material for: Methylmalonic acidemia triggers lysosomal-autophagy dysfunctions
Source: Cell Biosci. 2024 May 17;14:63. doi: 10.1186/s13578-024-01245-1 (PMC11102240; doi:10.1186/s13578-024-01245-1)
Supplement: Supplementary file 2 — Supplementary Material 2 [file 13578_2024_1245_MOESM2_ESM.docx]

**Supplementary Figures**

**Methylmalonic acidemia triggers lysosomal-autophagy dysfunctions**

**Michele Costanzo^1,2^*, Armando Cevenini^1,2^, Laxmikanth Kollipara^3^, Marianna Caterino^1,2^, Sabrina Bianco^1,2^, Francesca Pirozzi^1,2^, Gianluca Scerra^1^, Massimo D’Agostino^1^, Luigi Michele Pavone^1^, Albert Sickmann^3,4,5^, Margherita Ruoppolo^1,2^***

^1^ Department of Molecular Medicine and Medical Biotechnology, University of Naples Federico II, Naples, Italy

^2^ CEINGE–Biotecnologie Avanzate Franco Salvatore, Naples, Italy

^3^ Leibniz-Institut für Analytische Wissenschaften – ISAS – e.V., Dortmund, Germany

^4^ Department of Chemistry, College of Physical Sciences, University of Aberdeen, Aberdeen, Scotland, United Kingdom

^5^ Medizinische Fakultät, Medizinische Proteom-Center (MPC), Ruhr-Universität Bochum, Bochum, Germany

* Corresponding authors: michele.costanzo@unina.it; margherita.ruoppolo@unina.it

Department of Molecular Medicine and Medical Biotechnology, University of Naples Federico II, Via Pansini 5, 80131 Naples, Italy.

**Fig. S1.** Cell viability assays performed in MMA fibroblasts. A) Neutral Red (NR) uptake assay was performed normalizing NR absorbance values with paralleled crystal violet assay and reported as relative units (R.U.) in whisker plots; ns=not significant. B) MTT assay was performed normalizing MTT absorbance values with paralleled crystal violet assay and reported as R.U. in whisker plots; ns=not significant.


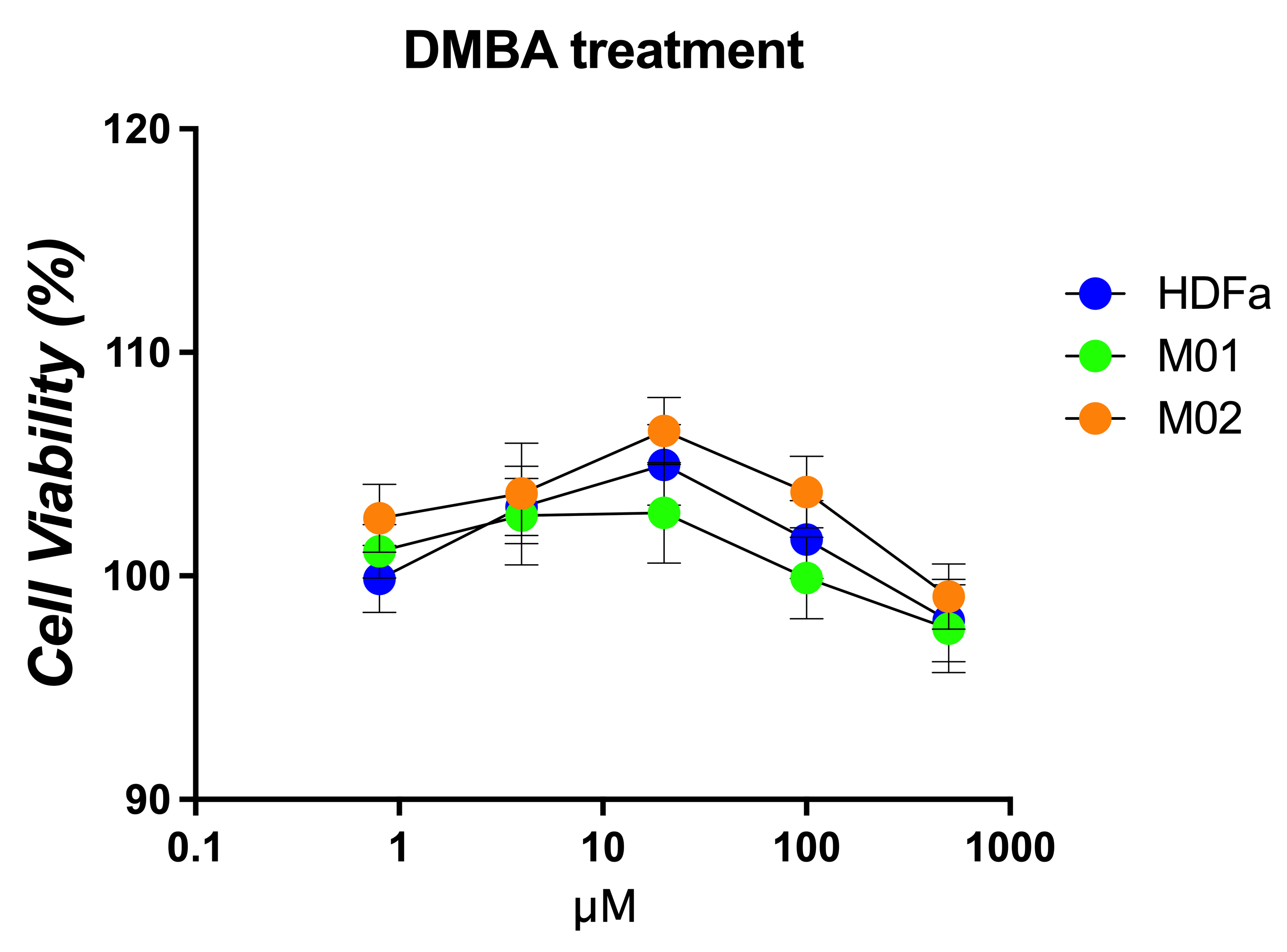


**Fig. S2.** Cell viability assay performed on HDFa and Mut0 fibroblasts using different DMBA concentrations to show non-toxic effects of higher concentrations of DMBA. The concentrations tested were 0, 0.8, 4.0, 20.0, 100.0, 500.0 µM and cell viability was monitored by crystal violet assay. Statistical analysis performed by one-way ANOVA showed no significant difference in the cell viability at the tested concentrations.
