# Supplementary material for: Methylmalonic acidemia triggers lysosomal-autophagy dysfunctions
Source: Cell Biosci. 2024 May 17;14:63. doi: 10.1186/s13578-024-01245-1 (PMC11102240; doi:10.1186/s13578-024-01245-1)
Supplement: Supplementary file 3 — Supplementary Material 3 [file 13578_2024_1245_MOESM3_ESM.docx]

**Supplementary Materials**

**Materials for proteomic analyses**

Following materials were purchased from Sigma-Aldrich, Germany: ammonium bicarbonate (NH_4_HCO_3_), anhydrous magnesium chloride (MgCl_2_), guanidine hydrochloride (GuHCl), iodoacetamide (IAA), complete mini EDTA-free protease inhibitor cocktail tablets and urea. Tris base was bought from Applichem Biochemica, Darmstadt, Germany. Sodium dodecyl sulfate (SDS) was purchased from Carl Roth, Karlsruhe, Germany. Dithiothreitol (DTT) was bought from Roche Diagnostics, Mannheim, Germany. Benzonase Nuclease (Purity > 99%), sodium chloride (NaCl) and calcium chloride (CaCl_2_) were purchased from Merck, Darmstadt. Sequencing grade modified trypsin was purchased from Promega, Madison, USA. Bicinchoninic acid assay (BCA) kit was bought from Pierce Thermo Fisher Scientific, Schwerte, Germany. iRT – indexed Retention Time kit was purchased from Biognosys AG, Switzerland. All chemicals for ultra-pure HPLC solvents such as formic acid (FA), trifluoroacetic acid (TFA) and acetonitrile (ACN) were purchased from Biosolve, Valkenswaard, the Netherlands.

**Filter-Aided Sample Preparation (FASP) of HEK 293 cells for DIA proteomics analysis**

HEK 293 WT, MUT-KO, and MUT-RES cells (1×10^6^ cells; n=5 per condition) were lysed with 150 µL of 1% SDS buffer containing 50 mM Tris-Cl, 150 mM NaCl, pH 7.8 with inhibitor protease mix. To degrade all nucleic acids (DNA and RNA), cell lysates were treated with 1% Benzonase plus 2 mM MgCl_2_ at 37°C for 30 min, and next centrifuged at 18,000 rcf at room temperature (RT) for 30 min ^1^. The clear supernatant was collected and protein concentration determined by BCA assay as per manufacturer’s instructions. Protein samples (50 µg) underwent reduction of disulfide bonds with 10 mM DTT at 56°C for 30 min, and carbamidomethylation with 30 mM IAA at RT for 30 min in the dark. Sample cleaning and proteolysis were performed using the Filter-Aided Sample Preparation (FASP) protocol with some changes ^2–4^. Briefly, carbamidomethylated cell lysates were diluted with freshly prepared 8 M urea in 100 mM Tris-HCl, pH 8.5 ^5^ and placed on a Nanosep centrifugal device (30 KDa, PALL). The devices were centrifuged at 13,900 rcf at RT for 20 min. All the following centrifugation steps were performed under similar conditions i.e., 13,900 rcf, RT, 15 min. To remove residual SDS, three washing steps were carried out using 100 µL of 8 M urea in 100 mM Tris-HCl, pH 8.5 and for the buffer exchange the devices were washed thrice with 100 µL of 50 mM NH_4_HCO_3_ buffer, pH 7.8. Then, 100 µL of digestion buffer comprising: trypsin 1:20 (w/w) enzyme-to-substrate ratio, 0.2 M GuHCl, 2 mM CaCl_2_ in 50 mM NH_4_HCO_3_ buffer, pH 7.8 were added to the concentrated proteins on the devices and incubated at 37°C for 14 h. Tryptic peptides were recovered by centrifugation followed by two consequent washing steps with 50 mM NH_4_HCO_3_ buffer, pH 7.8 and ultra-pure water. The peptide mixtures were acidified to pH < 3 with 10% TFA and desalted with C18 solid phase extraction cartridges (SPEC; 4 mg, Varian) according to the manufacturer’s instructions. Finally, the eluted peptides were vacuum-dried and resolubilized in 0.1% TFA, and digestion efficiency controlled on a Monolithic-HPLC as described previously ^6^.

**Amino acid analysis and high-pH reversed-phase HPLC fractionation**

Peptide quantification was performed by amino acid analysis (AAA) as previously described by Cohen et al. ^7^ and Shindo et al. ^8^. A “master mix” was generated by combining 1 µg of peptides (based on AAA) from each HEK 293 cell line and their corresponding replicates (n=15) resulting in 15 µg of pooled sample.

The “master mix” was dried in SpeedVac, resolubilized in 10 mM ammonium formate, pH 8.0 and fractionated by reversed-phase chromatography (RP-HPLC) at pH 8.0 on a Biobasic column (C18, 0.5×150 mm, 5 µm particle size) using an UltiMate 3000 LC system (both Thermo Scientific, Germany) with buffer A: 10 mM ammonium formate, pH 8.0; and B: 84% ACN in 10 mM ammonium formate, pH 8.0. Peptides were loaded onto the column with buffer A at a flowrate of 12.5 µL/min and separation was carried out using the following gradient: 3% B for 10 min, 3-38% B in 55 min, 38-95% B in 5 min, 95% B hold for 5 min, 95%-3% B in 5 min and finally re-equilibrate the column with 3% B for 20 min. In total 16 fractions were collected at 1 min intervals from min 5 to 70 in a concatenation mode, dried and stored at –40°C until further use.

**Spectral library generation and DIA-MS data analysis**

The acquired DDA MS data of the 16 high-pH fractions were processed together with Proteome Discoverer (PD) 1.4 (Thermo Scientific, Germany) software ^4^. Database searches were performed against the human UniProt database, (downloaded on 23^rd^ of July 2018, containing 20,734 target sequences including iRT sequence ^9^ using Mascot ^10^ and Sequest ^11^ algorithms. The search parameters were identical for both algorithms i.e., precursor and fragment ion tolerances of 10 ppm and 0.02 Da for MS and MS/MS, respectively; trypsin as enzyme with a maximum of 2 missed cleavages; carbamidomethylation of Cys as fixed modification and oxidation of Met as variable modification.

The data analysis of all DIA runs including the “scouting method” measurement was performed with Spectronaut Pulsar software (version 12.0.20491.8.23937). To generate the spectral library, the same human Uniprot database and the .msf PD 1.4 output file (as mentioned above) were uploaded in Spectronaut as per the recommended settings including 1% FDR and search engine rank 1 on the PSM level (from PD). Next, all DIA raw files were processed using the Spectronaut default settings i.e., MS1 and MS2 filtering, extracted ion chromatogram (XIC) extraction, calibration and identification. The settings under quantification were also set to default except with minor changes. The Min and Max values of Major Group Top N values were set to 2 and 4, respectively; Proteotypicity Filter was set to Only Proteotypic; Data Filtering was set to Qvalue (type Qvalue sparse), Cross Run Normalization set as True, Normalization Strategy was set as Global Normalization and Normalize on set to Median.

**Neutral-Red uptake, MTT and Crystal Violet Assays**

For both the MTT and NR uptake assays the culture medium was removed from the well and replaced with a fresh medium containing 0.5 mg/mL of MTT (3-(4,5-dimethylthiazol-2-yl)-2,5-diphenyltetrazolium bromide) or 0.33 mg/mL of the NR solution (both Sigma-Aldrich). Cells were incubated with the reagents for 2 h at 37 °C and washed with PBS. Then, for MTT a solution of 1 N hydrogen chloride-isopropanol (1:24, v/v) was pipetted to each well and mixed to dissolve the dark-blue formazan crystals formed; for NR a solution of acetic acid-water-ethanol (1:49:49, v/v/v) was added. Finally, the absorbance of each sample was read in a Perkin Elmer Enspire microplate reader at 570 nm or 540 nm for MTT or NR, respectively.

For crystal violet assay the culture medium was removed from the wells and, after two washes with PBS, replaced with crystal violet staining solution (CVSS) [0.5% (w/v) crystal violet in H_2_O/CH_3_OH 4:1 (v/v)]. CVSS was also added to wells without cells. The plate was incubated for 20 min at RT under gentle shaking on a bench rocker, washed four times with PBS and air-dried for 2 h under a chemical hood. 200 µL of methanol were added to each well and the plate was incubated with its lid on for 20 min at RT under gentle shaking. The absorbance of each sample was read at 570 nm in the Enspire plate reader. The average absorbance at 570 nm of processed wells without cells was considered background and subtracted from cell sample values.

**Antibodies**

The primary antibodies used for WB were the following: MUT (sc-390978, Santa Cruz Biotechnology, Dallas, TX, USA), Stathmin1 (ab52630, Abcam, Cambridge, UK), LAMP1 (SAB4700416, Sigma-Aldrich), LAMP2 (AB25631 Abcam), LC3 (NB100-2220, Novus Biologicals, Centennial, CO, USA), p62/SQSTM1 (sc-28359, Santa Cruz Biotechnology), TFEB (#4240, Cell Signaling Technology, Danvers, MA, USA), EGFR (#4267, Cell Signaling Technology), β-actin (ab8226, Abcam), α-tubulin (T6074, Sigma-Aldrich), histone H2A (ab18975, Abcam).

Antibodies used for immunofluorescence were the following: mouse anti-CD107a (LAMP1) (SAB4700416 clone H4A3) from Sigma-Aldrich; rabbit anti-Calnexin (SPC-108) from StressMarq Biosciences Inc. (Victoria, Canada); rabbit anti-LC3B (D3) (#3868, Cell Signaling Technology); anti-mouse (A11029, A11030) and anti-rabbit (A11034, A11035) Alexa-Fluor 488 and 546-conjugated secondary antibodies, respectively (Thermo Fisher Scientific).

**RT-qPCR**

Quantitative RT real-time PCR was performed on mut0 cells, after harvesting by trypsinization and pelletting by centrifugation. Total RNA was extracted from cell pellets using a RNeasy Mini Kit (Qiagen, Hilden, Germany). In particular, 0.5 µg of RNA were reverse-transcribed using SuperScript™ VILO™ MasterMix (Thermo Fisher Scientific, Bremen, Germany). RT-qPCR was performed in a 7900 Real-Time PCR Thermal Cycler System using a SYBR Select Master Mix (Applied Biosystems, Monza, Italy). Gene expression levels of *LAMP1*, *LAMP2* and *ATP6V1H* were normalized to *β-actin* (*ACTB*) and calculated using the 2^−∆∆Ct^ method. Three independent experiments were carried out. The sequences of the primers are reported as follows:

| **ACTB** | Forward | 5’-CGACAGGATGCAGAAGGAGA-3’ |
| --- | --- | --- |
|  | Reverse | 5’-CTGCATACTCCTGCTTGCTG-3’ |
| **ATP6V1H** | Forward | 5’-CATTCTTGCAGCATTTCGTAAC-3’ |
|  | Reverse | 5’-TTCTGCTGTTCCAAGTTCTCC-3’ |
| **LAMP1** | Forward | 5’-AGAGGAAGGACAACACGACG-3’ |
|  | Reverse | 5’-TGGAAGAGCAGGACGGTGG-3’ |
| **LAMP2** | Forward | 5’-ACTTAGACTCAATAGCAGCACC-3’ |
|  | Reverse | 5’-CTGAAAACGGAGCCATTAACC-3’ |

**References**

1. Costanzo, M. *et al.* Proteome data of neuroblastoma cells overexpressing Neuroglobin. *Data Br.* **41**, 107843 (2022).

2. Wiśniewski, J. R., Zougman, A., Nagaraj, N. & Mann, M. Universal sample preparation method for proteome analysis. *Nat. Methods* **6**, 359–362 (2009).

3. Manza, L. L., Stamer, S. L., Ham, A.-J. L., Codreanu, S. G. & Liebler, D. C. Sample preparation and digestion for proteomic analyses using spin filters. *Proteomics* **5**, 1742–5 (2005).

4. Costanzo, M. *et al.* Dataset of a comparative proteomics experiment in a methylmalonyl-CoA mutase knockout HEK 293 cell model. *Data Br.* **33**, 106453 (2020).

5. Kollipara, L. & Zahedi, R. P. Protein carbamylation: In vivo modification or in vitro artefact? *Proteomics* **13**, 941–944 (2013).

6. Burkhart, J. M., Schumbrutzki, C., Wortelkamp, S., Sickmann, A. & Zahedi, R. P. Systematic and quantitative comparison of digest efficiency and specificity reveals the impact of trypsin quality on MS-based proteomics. *J. Proteomics* **75**, 1454–1462 (2012).

7. Cohen, S. A. & Michaud, D. P. Synthesis of a Fluorescent Derivatizing Reagent, 6-Aminoquinolyl-N-Hydroxysuccinimidyl Carbamate, and Its Application for the Analysis of Hydrolysate Amino Acids via High-Performance Liquid Chromatography. *Anal. Biochem.* **211**, 279–287 (1993).

8. Shindo, N. *et al.* Separation of 18 6-Aminoquinolyl-carbamyl-Amino Acids by Ion-Pair Chromatography. *Anal. Biochem.* **249**, 79–82 (1997).

9. Escher, C. *et al.* Using iRT, a normalized retention time for more targeted measurement of peptides. *Proteomics* **12**, 1111–1121 (2012).

10. Perkins, D. N., Pappin, D. J., Creasy, D. M. & Cottrell, J. S. Probability-based protein identification by searching sequence databases using mass spectrometry data. *Electrophoresis* **20**, 3551–67 (1999).

11. Eng, J. K., McCormack, A. L. & Yates, J. R. An approach to correlate tandem mass spectral data of peptides with amino acid sequences in a protein database. *J. Am. Soc. Mass Spectrom.* **5**, 976–989 (1994).
